# Group A Streptococcal Septic Hip Arthritis in a Child With Spastic Triplegic Cerebral Palsy

**DOI:** 10.5435/JAAOSGlobal-D-20-00228

**Published:** 2021-08-05

**Authors:** Supriya Singh, Jacob Davidson, Timothy Carey, Michelle Barton Forbes, Megan Cashin

**Affiliations:** From the Division of Paediatric Orthopedic Surgery, London Health Sciences Centre (Dr. Singh, Mr. Davidson, Dr. Carey); the Division of Infectious Diseases, Department Pediatrics, London Health Sciences Centre (Dr. Barton Forbes); and the Department of Orthopedic Surgery, Janeway Children's Health and Rehabilitation Centre (Dr. Cashin).

## Abstract

Reports of septic hip arthritis in children with cerebral palsy are exceedingly rare. This case report describes a 10-year-old boy with spastic triplegic cerebral palsy (Gross Motor Functional Classification System), who presented with fever and irritability. This case highlights the difficulties in diagnosing septic joint arthritis in patients with cerebral palsy who are nonverbal and have limited mobility. A high index of suspicion is necessary in this population when presented with fever and new limitations in mobility.

Pediatric septic arthritis can be a life-threatening and limb-threatening condition. Accurate and timely diagnosis is important in the treatment of septic arthritis to minimize long-term sequelae and optimize outcome. Children with cerebral palsy who already have preexisting joint concerns may pose a challenge to early detection of septic arthritis. To the best of our knowledge, there is a paucity of literature addressing septic arthritis in patients with cerebral palsy.^1^

We present a case of acute Group A Streptococcus (GAS) septic arthritis of the hip in a child with nonverbal cerebral palsy with lower extremity spasticity and contractures.

## Case Report

A 10-year-old fully immunized boy with a history of posthemorrhagic hydrocephalus with ventriculoperitoneal shunt, well-controlled seizures on anticonvulsant therapy, global developmental delay, and spastic triplegic cerebral palsy (CP), presented to the emergency department with irritability, decreased mobility, and fever (39.3°C). The patient's mother noted that his baseline gross motor function had changed in that he was unable to crawl at home. Before onset of symptoms, this was his primary mode of independent mobility at home; otherwise, he ambulated with a full support walker at school and used a wheelchair in the community. His mother noted that the patient seemed to localize symptoms to the left leg. No preceding trauma was observed. Both the patient and his siblings had had upper respiratory symptoms in the preceding 2 weeks, and the patient's respiratory symptoms had resolved within a week of onset. No history of drowsiness, vomiting, or seizures was observed.

**Figure 1:**
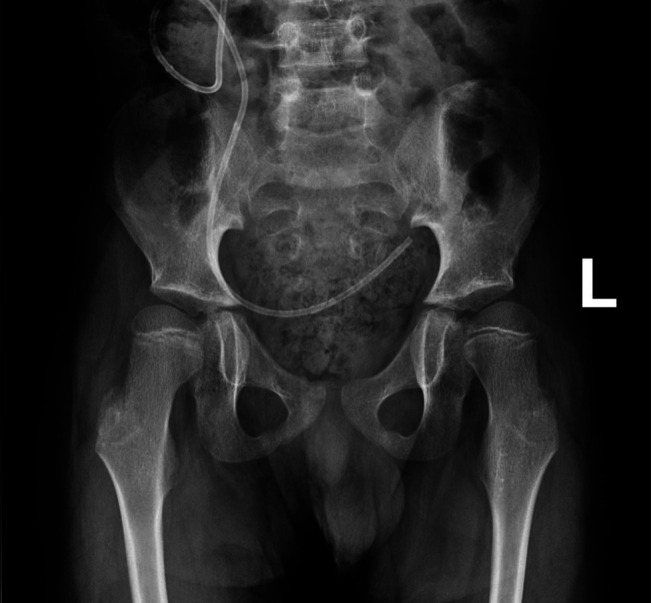
Prehospital admission anterior-posterior radiograph of pelvis indicating preexisting hip subluxation with a Reimers migration index of 40%.

This patient was followed by the pediatric orthopaedic team on a regular basis for management of the musculoskeletal manifestations of cerebral palsy, including lower extremity spasticity and contractures. At baseline, he functioned at a Gross Motor Functional Classification System (GMFCS) level of 4 (see Appendix 1, http://links.lww.com/JG9/A137). Previous hip examinations noted hip adduction and flexion contractures. Previous radiographs performed 24 months before his admission demonstrated left hip uncovering with a Reimers migration index of approximately 40% (Figure 1).

At presentation, his temperature was 39.3°C, the heart rate was 120 beats per minute with a capillary refill <2 seconds, and the blood pressure was 127/73 mm Hg. He was in no respiratory distress, and his chest was clear. No notable meningism or tenderness over palpation of shunt was observed, and irritability was only noted when his lower limbs were handled. Notable findings were limited to the musculoskeletal examination. The left knee and ipsilateral hip were held fully flexed, and the limb was adducted. There was no obvious swelling or discoloration of overlying skin of any of joints in either lower limb. The patient seemed more uncomfortable with passive movement of left lower limb.

**Figure 2:**
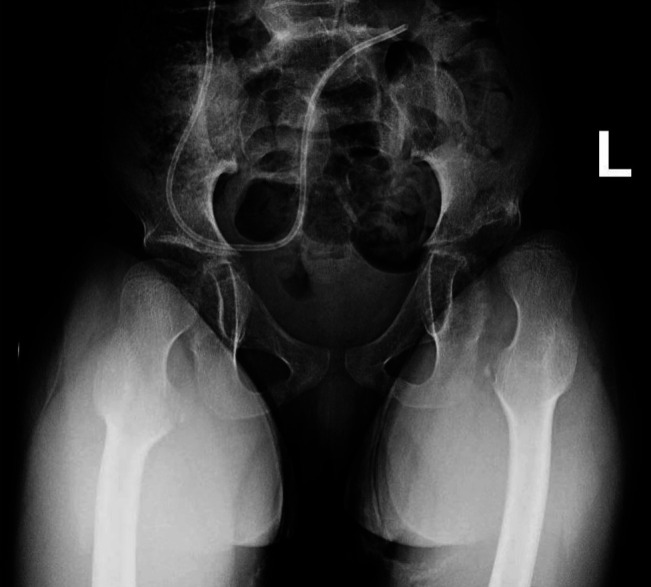
At time of hospital admission, anterior-posterior radiograph of the pelvis revealing worsening left hip subluxation with a Reimers migration index of 80%.

Initial investigations revealed a normal total white blood cell count (6.2 × 10^9^/L) with a neutrophil count of 4.7 × 10^9^/L, an elevated C reactive protein (CRP) of 176 mg/L, and an erythrocyte sedimentation rate of 85 mm/hr. Radiographs of the left lower extremity revealed no acute fractures while pelvis radiographs revealed that the left hip was subluxated, with a notable progression in femoral head subluxation from preadmission radiographs (Reimers migration index of 80%) (Figure 2).

Based on the patient's fevers, elevated inflammatory markers, and changes in behavior leading to irritability and inability to crawl, the patient was admitted for pain control and further investigation, including an ultrasonography-guided aspiration of the left hip. At this time, the working differential diagnosis for his musculoskeletal findings included septic hip arthritis and/or osteomyelitis of femur, transient synovitis of the hip, or symptomatic hip subluxation from cerebral palsy. Blood cultures grew GAS (*Streptococcus pyogenes*) within 20 hours of collection on day 1 of hospitalization and again on day 2 of hospitalization. His hip aspirate revealed a white cell count of 33.75 × 10^9^/L, with 93% neutrophils and gram-positive cocci seen on microscopy, and the cultures were subsequently confirmed to be GAS. Within a few hours of the preliminary aspirate results, the patient was taken to the operating theater for an open débridement and irrigation of the left hip joint. Intraoperatively, the hip capsule was distended and under tension, and on decompression, a large amount of serosanguinous fluid was expressed which also grew GAS. The cartilage on the femoral head was intact. A drain was placed in situ to allow postoperative drainage.

Postoperatively, treatment included administration of intravenous Cefazolin. Although there was mild improvement in pain, fevers persisted. CRP rose to 395 mg/L on postoperative day 1. The white blood cell count remained normal, and repeat blood cultures were negative.

Given the persisting temperature spikes, the patient underwent further investigations including a lumbar puncture in search for another focus of infection and was empirically switched from Cefazolin to Ceftriaxone and Vancomycin at meningitic dosing. The latter was discontinued after cerebrospinal fluid analysis and culture excluded meningitis. Urine culture was negative. Respiratory viral panel returned negative for common viral pathogens (influenza, respiratory syncytial virus, parainfluenza, adenovirus, and enterovirus). Lung fields were clear on chest radiograph. An echocardiogram failed to identify findings concerning for endocarditis or myopericarditis.

Two weeks after the initial irrigation and débridement, reaccumulation of hip joint effusion was entertained based on the persistent fevers, recurrent hip irritability, and rising CRP. Magnetic resonance imaging (MRI) was done to rule out osteomyelitis and showed a recurrent effusion. Based on the patient's clinical symptoms and MRI results of persistent joint effusion, he was brought back to the operating theater for repeat irrigation and débridement. Intraoperative hip fluid cultures were sterile.

**Figure 3:**
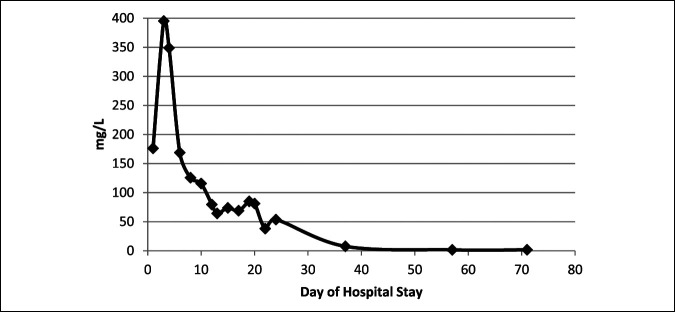
Graph showing the CRP (C-reactive protein) trend during hospital admission and postoperative course.

After this second irrigation and débridement, the patient's clinical picture improved markedly. He allowed free range of motion of both lower limbs without crying, his fevers had resolved, and his CRP had normalized within 2 weeks of this operation (Figure 3). One month after admission to hospital, the patient was discharged with his clinical picture returning to baseline.

**Figure 4:**
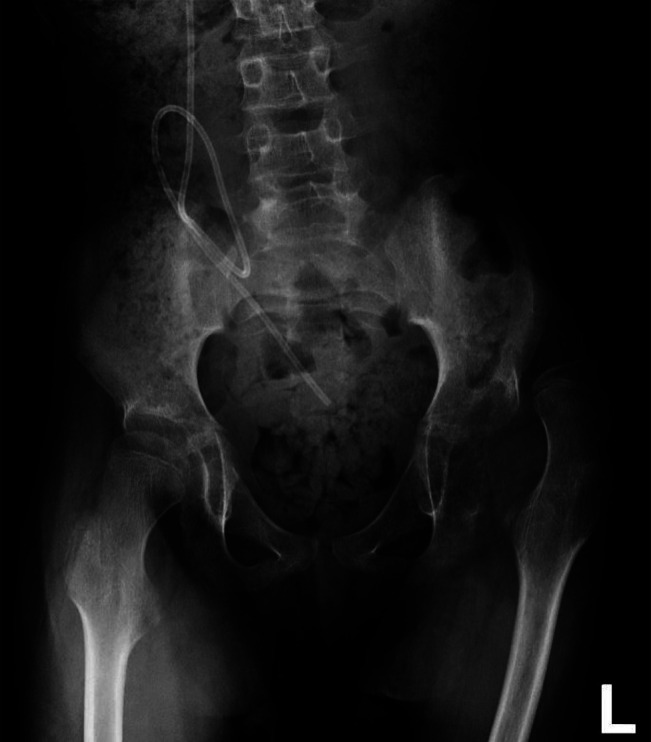
Six-week postoperative anterior-posterior radiograph after the second surgery of the pelvis revealing a left hip dislocation.

**Figure 5:**
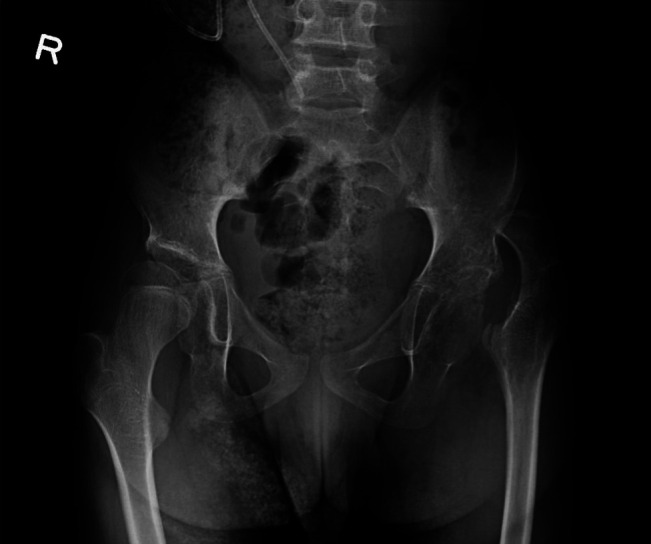
One-year postoperative anterior-posterior radiograph after two irrigation and débridement surgeries of left hip joint for septic hip arthritis revealing a high riding left hip dislocation.

The patient's final diagnosis was Group A Streptococcal septic hip arthritis. He required two operations to gain source control over a 2.5-week period. He completed a 3-week course of intravenous Ceftriaxone and once he was afebrile received an additional 3 weeks of high-dose Cephalexin to ensure he had a full 4 weeks of antibiotic therapy from his second joint irrigation. At the 6-week mark, his CRP was normal and his left hip was nonpainful. He had returned to his baseline preoperative gross motor function. Radiographs showed no evidence of osteomyelitis but did reveal progression of the preoperative hip subluxation to complete dislocation (Figures 4 and 5).

It should be noted that throughout the patient's hospital stay, a multidisciplinary team approach was used for this patient. In addition to the pediatric orthopaedic team, the pediatric medicine team, the infectious disease team, and the pediatric neurology team were involved in treating this patient.

## Discussion

This case highlights the challenges in diagnosis and treatment of a septic hip in a child with cerebral palsy, GMFCS 4. There is minimal literature regarding septic joints in patients with cerebral palsy.^2^ In this patient population, the clinical diagnosis may be challenging given expressive language limitation, as well as preexisting range of motion limitations from contractures or joint subluxation, and preexisting gait abnormalities in ambulators. Clinical examination is even more challenging in those unable to ambulate (ie, those with higher GMFCS levels). Suspicion for a diagnosis of septic joint arthritis should seriously be entertained in this population, if caregivers note new mobility limitations in the context of high fevers, especially when an obvious focus is not apparent given that delayed diagnosis can have notable consequences.

Single joint involvement, the presence of elevated inflammatory markers, and documented bacteremia, especially with organisms commonly associated with musculoskeletal infections, should heighten suspicion for septic arthritis.^3^ Ultrasonography imaging or MRI, especially if there are also concerns for osteomyelitis, allows detection of an effusion, which can then be aspirated for conclusive determination of infection.

GAS is a well-recognized cause of septic arthritis and may arise from a hematogenous source or from local spread from an overlying skin or soft-tissue infection.^3^ In our patient in the absence of a skin or soft-tissue source, it is more likely that the preceding viral upper respiratory tract infection may have facilitated entry of GAS across inflamed upper respiratory mucosa into his circulation causing bacteremia. Once bacteremic, a septic arthritis may have developed as a result of seeding of an already abnormal joint. Although septic arthritis was confirmed within a day of admission after gram-positive cocci were seen on gram stain of joint fluid and was promptly irrigated, the patient's course was complicated by a marked inflammatory response with climbing CRP and persisting fevers. The persisting fever prompted an extensive workup for disseminated GAS infection. The patient's underlying history of recurrent pneumonia and preexisting shunt hardware made it necessary to exclude central nervous system and respiratory sources. The high fevers and multiple positive blood cultures prompted an echocardiogram to exclude infective endocarditis. This patient's preexisting joint limitations and recent surgical procedure were believed to be a plausible explanation for ongoing painful limitation of hip mobility and created delays in clinical detection of reaccumulating joint effusion. In addition, the complexity of the patient's medical history, which placed our patient at risk for central nervous system and respiratory infections, caused us to shift focus from the joint as an ongoing source of sepsis. The joint was reimaged as a part of the workup to exclude bony extension, and it is this MRI that allowed the detection of reaccumulating joint fluid and facilitated joint irrigation. The fluid obtained intraoperatively in the second surgical irrigation and débridement was notably sterile, and therefore, it is more likely that the large joint collection was driving a noninfectious inflammatory response. GAS is noted to be able to incite an exaggerated inflammatory response that can continue even after bacterial clearance.^4^

Our case emphasizes that it is important to reimage the affected joint early if there is persistent fever or poor CRP response in a CP patient who is nonverbal and in whom hip examination is difficult or hard to conclusively determine if pain is related to postoperative pain and/or preexisting contractures limiting movement.

Interestingly, in this case, at presentation the preexisting hip subluxation had worsened. A Reimers migration index had increased from 40% to 80%, with frank dislocation noted postoperatively (Figures 1, 2, and 4, respectively). Postoperatively, the hip was dislocated, suggesting that infection may have led to hip instability or that hip instability may have been worsened by surgical intervention that included capsulotomy. There is minimal literature available in the cerebral palsy population discussing septic hip arthritis and long-term sequelae including hip instability.^5–7^ The rapid progression seen in this case has not been similarly reported to the best of our knowledge. In addition, 1-year postoperative pelvis radiographs show worsening of the hip dislocation (Figure 5), increasing the complexity of management for this patient and suggestive of rapidly progressive and worsening instability postoperatively and postinfection.

In the general pediatric population, long-term sequelae of septic hip arthritis include pain, postinfectious deformity leading to arthritis, limb length discrepancies, and possibly joint instability. These late sequelae seem to be rare events if patients are treated early with irrigation and débridement and appropriate antibiotic management. Baghdadi et al reviewed 14 children with septic hip arthritis who had onset before age 4. After examination of these patients' courses of treatment, they conclude that early recognition and treatment can prevent morbidity and disability from long-term sequelae of septic arthritis. Ten of the 14 patients were treated early and had minimal pain and deformity including coxa magna in the long term. One of the four patients not treated with early surgical intervention developed joint instability. This was attributed to the notable deformity caused by the lack of treatment to this septic hip. This patient had destruction of the femoral head and neck and as such had no effective articulation of the hip joint and was classified as unstable. Most patients in this case report had early treatment and as such had minimal residual deformity or mild coxa magna.^6^ A study published in Italy by Campagnaro et al evaluated long-term sequelae of 73 hips with septic arthritis. Sixteen percent had poor outcomes with the most notable sequelae being hip instability and joint dysfunction.^8^

The long-term effects of septic hip arthritis depend on the age of occurrence whether there was delayed initiation of antimicrobial treatment and the shape of the hip joint at skeletal maturity.^5^ For example, in infants under age 6 months old who have septic hip arthritis, delayed, inadequate, or no treatment can lead to complete destruction of the proximal femoral epiphysis and consequently to an unstable hip joint. On the other hand, untreated septic arthritis in older children is most likely to lead to a stiff hip joint and ankylosis.^7^ Given that our patient is older, we would have predicted joint stiffness rather than instability. This deviation from expected course may indicate that perhaps patients with cerebral palsy have a different pathophysiology or suggests that perhaps repeated surgical intervention contributed to instability.

In conclusion, this case highlights the difficulties in diagnosing and managing septic joint arthritis in pediatric patients with cerebral palsy. These difficulties include the atypical features of the clinical presentation, the possibility of postinfectious progression of hip subluxation, and, in this particular case, the resistant nature of the inflammatory response related to the infection. Septic arthritis should be entertained in cerebral palsy patients who present with fever and new limitation of joint movement, especially if there is associated bacteremia.
